# Experience of the Sudanese doctors in surgery of conjoined twins

**DOI:** 10.1093/jscr/rjad293

**Published:** 2023-06-05

**Authors:** Isam Ahmed Abdeljaleel Taha, Mohamed Abdalaal Hussin Helali, Sami Mohamed Alamin Taha, Ali Hamad Mahmmoud Hamad, Shaima Osman Mohamed Ali Alaraby, Abdallah Elsiddig Dafallah, Mohammed Mahmmoud Fadelallah Eljack, Khabab Abbashar Hussien Mohamed Ahmed, Basil Abubakr Yagoub Ibrahim, Abdalrhman Hassan Ahmed Mohamed, Mohamed Yahia Ibrahim, Ishag Nadi Joseph Wisa

**Affiliations:** Department of Pediatric Surgery, National Ribat University Hospital, Khartoum, Sudan; Department of Pediatric Surgery, National Ribat University Hospital, Khartoum, Sudan; Department of Pediatric Surgery, Sidra Medicine, Doha, Qatar; Department of Pediatric Surgery, King Faisal Specialist Hospital and Research Center, Madinah, Saudi Arabia; Department of Pediatric Surgery, Ibn Sina University, Khartoum, Sudan; Department of Pediatric Surgery, National Ribat University Hospital, Khartoum, Sudan; Department of Community Medicine, Faculty of Medicine and Health Sciences, University of Bakht Alruda, Ad Duwaym, Sudan; Department of Medicine, Faculty of Medicine, University of Khartoum, Khartoum, Sudan; Department of Medicine, Faculty of Medicine, University of Khartoum, Khartoum, Sudan; Department of Pediatric Surgery, National Ribat University Hospital, Khartoum, Sudan; Department of Pediatric Surgery, National Ribat University Hospital, Khartoum, Sudan; Department of Pediatric Surgery, National Ribat University Hospital, Khartoum, Sudan

## Abstract

Surgical separation of conjoined twins remains one of the most unique and rewarding experiences in the field of pediatric surgery, bearing in mind that this decision is their best chance of survival. These are the first reported cases of successfully separating omphalopagus conjoined twins by the liver in Sudan. After an emergency cesarean section, 62-day-old term-conjoined twins were referred to our pediatric surgery center. Examination revealed well-appearing twins fused from the xiphoid to the umbilicus; imaging confirmed a fused liver with a separate portal and caval structures, necessitating surgical separation and closure, which was done successfully on subsequent hours with well tolerance and recovery discharged on day 21. The second case involved 21-day-old term-conjoined female twins who were fused from the xiphoid to the umbilicus and shared the same cord, as well as complete fusion of the liver with separate other vital organs. They were successfully separated and recovered well.

## INTRODUCTION

Throughout history, the separation of conjoined twins has remained one of the most difficult challenges in the field of pediatric surgery, owing to the technical and ethical complexity of the procedure, which is extremely risky and life-threatening in the majority of cases [1]. This complexity mainly depends on the position of the fusion and the shared internal organs involved [1, 2]. The most common forms are thoracopagus, omphalopagus, pyopagus, isciopagus and craniopagus [3].

The incidence varies between one in 50 000 and one in 20 000 live births with female commonality [3, 4]. The diagnosis can be made from 12 weeks gestation through prenatal ultrasonography, while at 20 weeks the anatomy and extent of the conjoined area can be identified [4].

Even though these twins appear theoretically divisible, any attempt to separate them may result in severe hemorrhage and hypovolemic shock, especially if done early in life. These are infrequent in the ischiopagus and pyopagus varieties [1].

Herein we report the first two cases in Sudan of Omphalopagus twins conjoined by the liver referred to our pediatric surgery center, separated successfully by our multidisciplinary team led by pediatric surgeons with a good outcome.

## CASE 1

A 62 days old, term-conjoined female twins were referred by ambulance to the pediatric-center police hospital, Sudan.

**Figure 1:**
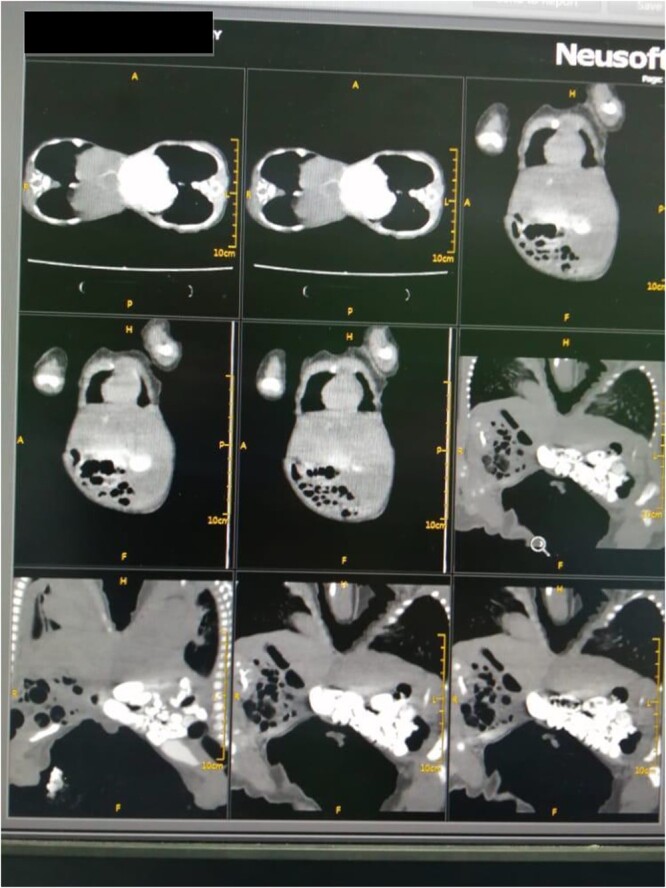
Abdominal computed tomography revealed a fusion of the liver through a large isthmus with normal liver and without vascular connections.

The mother was a 21-year-old female with a family history of multiple pregnancies, para three delivered uneventfully vaginally with a good outcome. Her pregnancy passed well with good antenatal care and early use of tonics, on 30 weeks the abdominal ultrasound revealed monochronic conjoined twins. At 40 weeks gestation, an emergency cesarian section was done due to labor pain, outcome was full-term, monochromic conjoined twins, cephalic, cried immediately and passed urine and meconium within 1st 24 h. On examination, both babies appeared well, not pale or jaundiced and not distressed, and weighed 2.4 kg for each one. They were fused from the xiphoid to the umbilicus with the used area covered by skin, and shared the same umbilical cord. Abdominal Computed tomography revealed a fusion of the liver through a large isthmus side by side with normal liver size and texture, no connections at the portal vein, normal vena cava and other structures (Fig. 1). other organs were entirely normal. Echocardiography showed that one twin has dextrocardia, a small restrictive Ventricular septal defect with a left to right shunt. So, the decision to elective surgical operation was made.

**Figure 2:**
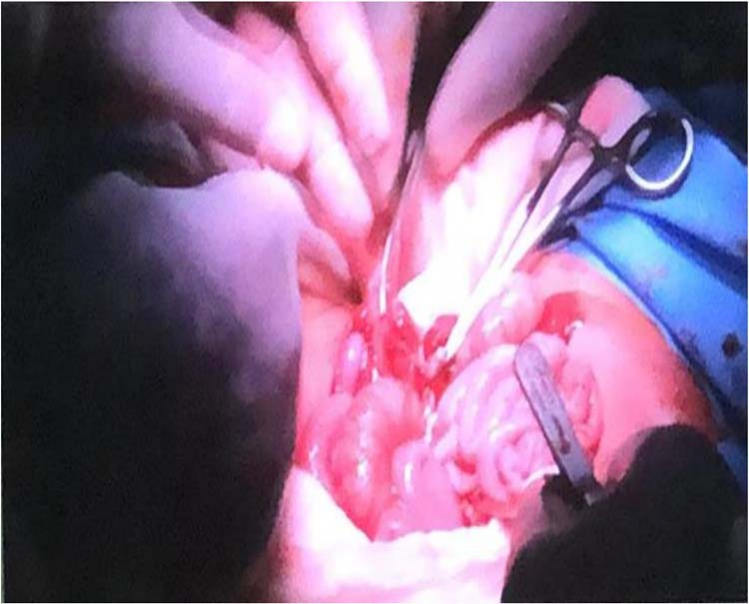
Connections within parietal peritoneum were identified and then carefully divided, followed by the connecting part of the liver.

After anesthetic preparation and central line insertion, both twins were placed on sterile sheets and then prepped anteriorly, Incision was made using monopolar and bipolar diathermy in the connecting bridge, skin and muscle were cut, connections within parietal peritoneum were identified, and then carefully divided, followed by the connecting part of the liver (Fig. 2), after two and half hours of surgery the twins were finally separated and other twin was taken to another table.

The abdominal wall was closed with Polydioxanone suture (PDS) sutures without mesh, and they were intubated before being transferred to the intensive care unit. Both babies were extubated within 2 days, and antibiotics were administered for another 21 days. On day 4, oral feeding began with good tolerance. Both babies gained weight and were discharged on day 25. They were later seen at the referring clinic after 2 weeks with good healing and well-being.

## CASE 2

A 21 days old, term-conjoined female twins were referred by ambulance to the pediatric-center police hospital, Sudan.

The mother is a primigravida of 24 years old with a positive family history of multiple pregnancies. Her pregnancy was uneventful, with regular antenatal care and tonic use; however, an abdominal ultrasound at week 13 revealed multiple pregnancy. Due to liquor drainage, she had an emergency cesarean section at 38 weeks; the outcome was term monochronic, conjoined twins, breech cried immediately and passed urine and meconium within the first 24 h. On examination, both twins appear healthy, not pale or jaundiced, fused from the xiphoid to the umbilicus and covered by skin, sharing the same cord with a combined weight of 4.5, twin A 2.5 kg and twins B 2 kg.

**Figure 3:**
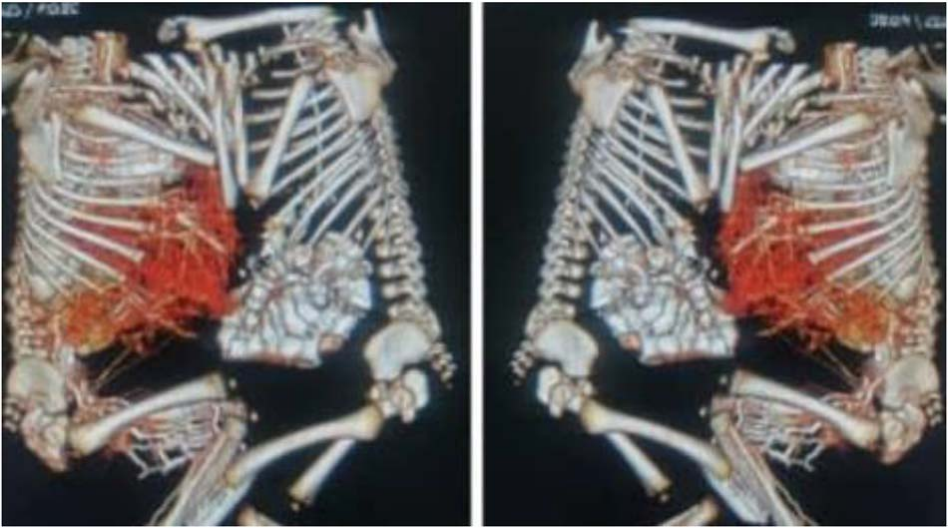
CT scan with contrast of case 2 showed omphalopagus type of conjoined twins with fusion of anterior aspect of the livers and separate other vital organs.

A computed tomography scan revealed ventral omphalopagus-xiphoid union of conjoined twins with separate vital organs and signs of fusion of the anterior aspect of the livers (adhesion), as well as one lower limb arteriovenous malformation (AVM) (AV fistula) seen extending from external iliac major vessels in both with difference in affected side (Fig. 3). Both twins’ echocardiography scans were completely normal. Both the complete blood count and the liver function test were normal.

**Figure 4:**
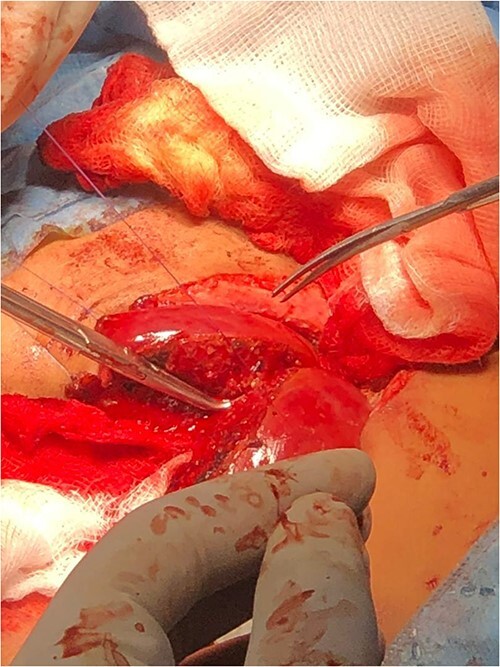
Separation of the shared liver in case 2.

Following the placement of central lines, the two babies were anathesized and stabilized with vital sign monitoring and fluid adjustments. Then, using monopolar and bipolar diathermy, incisions and flaps were made until the peritoneum, where complete fusion of the liver with a sinusoidal connection was discovered. The connected surface area is ⁓8 by 6 cm, primarily in the caudal lobe, whereas twins B has one lobe liver and one gall bladder (Fig. 4). The xiphoid process, sternum, pleura and pericardium were all visible in the patient and were successfully separated. To avoid the occurrence of compartment syndrome, the abdomen was left as a shallow cavity with only skin closed as a neonatal hernia, then hemostasis was secured via PDS sutures and drains were placed, admitted to neonatal intensive care unit and received blood and fresh frozen plasma. The patients recovered well from anesthesia on the second day, were taken off mechanical ventilation with normal vital signs, and began Nasogastric tube tube feeding 48 h later. Patients began to gain weight and were discharged the next day to be seen at a referral clinic.

## DISCUSSION

The history of conjoined twins’ dates back to as early as 80 BC, with a stone, craved depicting pygopagus twins discovered in Fiesole Italy. The earliest known attempt to separate conjoined twins took place in Armenia in 945 AD, when a separation of the ischiopagus twin from his dead brother was done, however, he died 3 days later [5].

The first well-reported case of successful surgical separation of conjoined twins was by Farius in 1689 [2, 6].

Conjoined twins are monozygotic, monoamniotic and monochorionic twins. The condition is rare and has an incidence ranging from one in 50 000 to one in 200 000 per live birth, with only 50% being live born [5, 7]. It is estimated that 35% will not survive beyond 24 h, making only 18% of conjoined twins survive [7–9]. The mortality rate of 33% for those surviving beyond the neonatal period and undergoing surgical intervention. Factors predicting mortality include prematurity, extremely low birth weight and female sex [8].

The conjoined twins are classified into eight types, with five ventral types and three dorsal types. The most common variant is Thoracopagus/thoraco-omphalopagus, with omphalopagus incidence ranging from 18 to 32%, making it the second most common type [5, 9, 10].

According to the literature, there are no known genetic or environmental causes of this condition, with some studies suggesting assisted reproduction techniques and maternal griseofulvin use during pregnancy as possible associated factors [11, 12].

The omphalopagus subtype is characterized by the fusion of the upper abdomen, from the lower chest to the umbilicus, with heart fusion characteristically absent. Over 80% will have fused livers with exomphalos, a conjoined biliary tract in 25%, 16% will have a share upper foregut and up to one-third will have intestines joining usually at Meckel’s diverticulum and share terminal ileum and colon [5, 7, 13].

Prenatal diagnosis can be made as early as 12 weeks using fetal Ultrasound (USS). Other investigations that can be used are Fetal echocardiography for detailed cardiac assessment and magnetic resonance imaging as an alternative technique for overall fetal assessment [4, 5]. Prenatal diagnosis is important for decision-making, including the decision to terminate the pregnancy, determining the mode of delivery and making plans for surgical intervention [7]. Both of our cases had good antenatal care, with an abdominal ultrasound at 30 and 13 weeks revealing monochorionic conjoined twins in the first and second cases, respectively. An elective cesarean section was performed at 40 weeks for the first set of twins, while the second twins was delivered by an emergency cesarean section at 38 weeks.

The omphalopagus conjoined twins have the best chance of survival if the surgical separation was done successfully [3, 13]. However, the separation of the omphalopagus type may be challenging and complex. The success of surgery depends on various factors including the extent of organ sharing, the presence of anomalies, the presence of cross circulation and the expertise of the surgeon. Hence, it requires meticulous pre-operative planning and investigations as well as adequate post-operative care and follow-up to reach optimal outcomes, as it carries risks for hemorrhage and hemodynamic instability, vascular thrombosis and biliary complications [3, 8, 10, 13]. In the first case, both twins were investigated using USS of the abdomen to delineate the internal organ fusion extent, with only shared livers noted. An echocardiogram was also done to assess both twins’ hearts, which revealed no fusion, but the L twin heart showed dextrocardia with a small restrictive Ventricular septal defect and a left to right shunt. In the second set of twins, A computed tomography scan was done, revealing ventral omphalopagus-xiphoid union of conjoined twins with separate vital organs and signs of fusion of the anterior aspect of the livers, as well as one lower limb AVM (AV fistula) seen extending from external iliac major vessels in both twins, no further investigations were done. To the best of our knowledge, this is the first case report of omphalopagus conjoined twins with such a vascular anomaly.

The timing for surgery is largely controversial. Most surgeons would prefer the ‘watch and wait approach’ and delay the surgical intervention till after the neonatal period, where it is done in elective setting. O’Neil found that survival rate is at 50% if surgery done after the first 4 weeks, and it increases up to 90% after 4 months of age [13, 14]. Villarreal *et al.* suggested operation at 10 months [8]. This allows for proper preoperative investigations and time for good planning of the task and details of the operation by the multidisciplinary team in charge.

Despite this, some cases require emergency surgery, this might be in cases such as when one twin is a threat to the life of the other (e.g. Complex Congenital Heart Diseases, sepsis, death), presence of severe malformation that threatens the life of twins and require intervention, substantial damage of the connecting bridge and when one of the twins have severe deformity that is incompatible with life and the other one have a good chance of survival [6, 7]. In our first case, Twin L had a complex cardiac condition, but did not necessitate emergency operation. The separation of the twins in an elective setting, when they presented 2 months after delivery. At operation, only shared liver was noted, with a totally separated biliary system and no intestinal fusion is seen. In the second case, operation was also done in an elective setting, and despite computed tomography (CT) scan showing only anterior fusion, the livers were found to be completely fused with sinusoidal connection. The need for liver transplantation should be put in mind when undergoing such operations, and mothers should be tested and prepared for partial liver transplantation should the liver separation fails [14]. The cardiac defect in the first case didn’t need an intervention, and the externa iliac AVM in the second case is set to be dealt later by vascular surgery department.

Various methods for closure of abdominal wall, including using a mesh, tissue expander, free flaps and skin grafts. The used method depends on the surgeon’s preference as well as the size of the defect [5, 14]. In the first case, we closed the abdomen is closed using tissues expander direct closure since the defect was small (5 × 5 cm). In the second case, abdomen was left as a shallow cavity, with only skin closed as a neonatal hernia, then hemostasis was secured via PDS sutures and drains were placed.

Despite limited facilities in Sudan, a multidisciplinary team approach with meticulous pre-operative assessment and post-operative care has led to a success in managing these two cases. The first set of twins were seen 39 days after operation at referral clinic, revealing healthy infants with normal development and growth.

## CONFLICT OF INTEREST STATEMENT

None declared.

## FUNDING

The study was self-funded.

## CONSENT

Written informed consent was obtained from parents after proper counseling.

## DATA AVAILABILITY

The data set used and/or analysed during the study are available from the corresponding author on reasonable request.
